# Nanoscale Localization Microscopy and Deterministic Lithography of Solid-State Quantum Emitters

**DOI:** 10.1021/acsphotonics.5c02864

**Published:** 2026-02-03

**Authors:** Sam G. Bishop, Hüseyin B. Yağcı, Rachel N. Clark, John P. Hadden, Anthony J. Bennett

**Affiliations:** † Translational Research Hub, 2112 Cardiff University, Cardiff CF24 4HQ, UK; ‡ School of Engineering, 2112 Cardiff University, Cardiff CF24 3AA, UK; § Quantum Engineering Technology Labs, H. H. Wills Physics Laboratory and School of Electrical, Electronic, and Mechanical Engineering, 1980 University of Bristol, Bristol BS8 1FD, UK

**Keywords:** microscopy, localization, quantum emitters, single photon, aligned lithography, nanostructures, nano-photonics

## Abstract

Quantum emitters (QEs) in the solid state can be spatially aligned with nanostructures to increase the photon collection efficiency and radiative emission rate. In many promising material platforms, these QEs are randomly positioned over the sample area, necessitating precise mapping of the emitter location and subsequent agile lithography aligned with the source. We have developed a programmable confocal microscope system to localize QEs with subwavelength precision, and subsequently accurately define nanostructures around the emitters. We show that repeated sampling of emitter location relative to alignment markers can account for sample drift and localize the emitter position within a few tens of nanometers. We demonstrate the deterministic enhancement of the collected photon intensity by up to 84% for emitters embedded in a micropillar.

## Graphical abstract

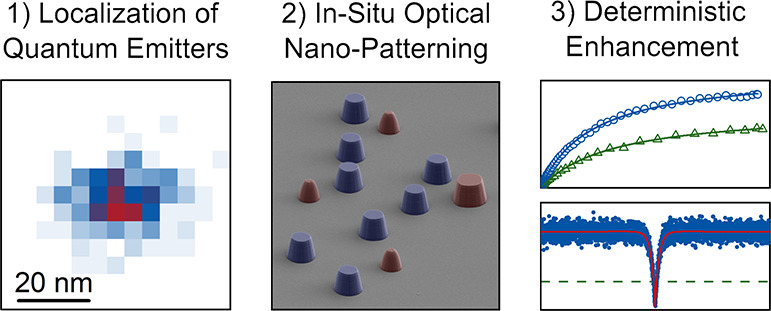

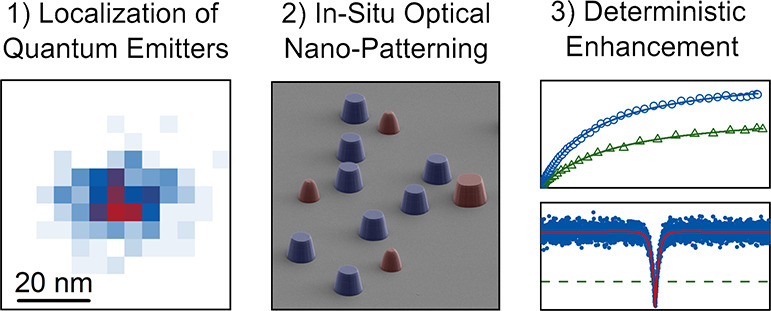

## Introduction

Quantum emitters (QEs) in the solid state offer a promising way to generate nonclassical light for tests of fundamental physics and for potential applications in quantum technology, such as quantum communication and quantum sensing.^1^ Room-temperature emission is especially sought after and has been observed in wide bandgap materials such as Si_x_N_y_,^2^ diamond,^3^ SiC,^4^ GaN,^5,6^ AlN,^7,8^ and hexagonal-BN.^9^ Common to many of these systems is the challenge of efficient photon extraction from a high-index material, which suffers from refraction and total internal reflection, into a collection lens of finite solid angle. Various strategies have been adopted to redistribute the emission into a reduced solid angle that is suitable for collection. For room-temperature QEs, which often have a phonon-broadened emission spectrum, such photon collection efficiencies must be broadband and spatially aligned to the QE.

“Bottom up” strategies to create QEs in regular arrays include ion implantation,^10,11^ femto-second laser writing,^12^ and prepatterning of the substrates.^13,14^ In some cases, it is possible to use sophisticated epitaxial techniques to simultaneously quantize the electronic states within a de Broglie scale and to achieve wavelength-scale photonic nanopillars that efficiently outcouple emission. Examples include cryogenic InAs/InP quantum dots in gold-catalyzed epitaxy^13^ and selective area epitaxy of InGaN on a patterned substrate, which can emit at elevated temperatures.^15^ Current methods of “bottom up” fabrication of QEs can lead to damage to the surrounding matrix, which may cause defects that fluoresce or degrade the coherence of the target emitter.

An alternative strategy is the “top down” method, where high-quality material with as-grown QEs in random positions in the plane, is precharacterized. The QEs are preselected for desired properties such as their emission energy and polarization, and their positions are noted relative to lithographic markers. Subsequent creation of nanostructures aligned with the QEs leads to a high yield of efficient and coherent devices. Such techniques have been demonstrated with InGaAs quantum dots at cryogenic temperatures^16–18^ using in situ low-temperature optical resists or ex situ electron beam lithography. The state-of-the-art uses wide-field fluorescence microscopy to localize the position of QEs relative to reflective alignment markers.^18^ This technique is suitable for QEs in optical cavities, where the excitation power density can be low.

Similar techniques have been adopted to integrate QEs with microantennas^19,20^ and solid-immersion lenses,^6,21–25^ which offer modest broadband efficiency enhancement and require approximately wavelength-scale positioning accuracy. More challenging is the use of nanostructures that may offer greater enhancements, or acceleration of the radiative emission via the Purcell effect, but require subwavelength positioning accuracy. Examples include plasmonic resonators^26^ and circular Bragg gratings.^27,28^

Several techniques have been developed to localize emitters below the Rayleigh diffraction limit, which is essential if nanostructures are to be precisely aligned to them. These include the use of higher-order optical modes in stimulated emission depletion microscopy (STED)^29^ and ground-state depletion microscopy.^30^ In general, for sparse, point-like emitters, fitting techniques can be used to localize the position of the source with an uncertainty $>\sigma /\sqrt{N}$ , where σ is the diffraction limit and *N* is the number of photons.^31^ In principle, longer integration times can increase *N* arbitrarily, improving the localization. However, in practice, sample drift and the repeatability of positioning limit the accuracy and precision with which this can be applied. Here, we show that repeated fast sampling of position, through measurements referenced to alignment markers, can overcome this bottleneck.

We present a versatile, easy-to-implement technique for nanoscale localization of solid-state QEs. We use a custom laser-scanning confocal lithography (LSCL) microscope and software package for both optical localization of QEs and deterministic patterning of spatially aligned dielectric microantennas. We demonstrate that cylindrical micropillars can enhance the light-extraction efficiency from room-temperature QEs in aluminum nitride over a wide spectral range. This highlights the versatility of the technique to pattern any manufacturable optical structures spatially aligned to QEs in the solid state, independent of the host material. The technique can be expanded for in situ nonlinear optical lithography^32^ for nanoscale manufacturing of optical devices spatially aligned to solid-state QEs.

## Laser Scanning Confocal Lithography

To localize the position of QEs and pattern spatially aligned nanostructures, we have developed a fully automated LSCL microscope. The LSCL microscope is illustrated in Figure 1a. The microscope exploits dual-axis galvanometric mirrors, a 0.9 numerical-aperture microscope objective, and a 4F optical relay system to displace the 520 nm excitation laser across the surface of the sample in *X* and *Y*. The system enables millisecond timescale per-pixel monitoring, control, and shuttering of the excitation laser, which is used for both imaging and polymerization of photosensitive resists on the sample surface. The same laser is used to avoid chromatic aberrations and misalignment between the localization of QEs and patterning of the photoresist.

**Figure 1:**
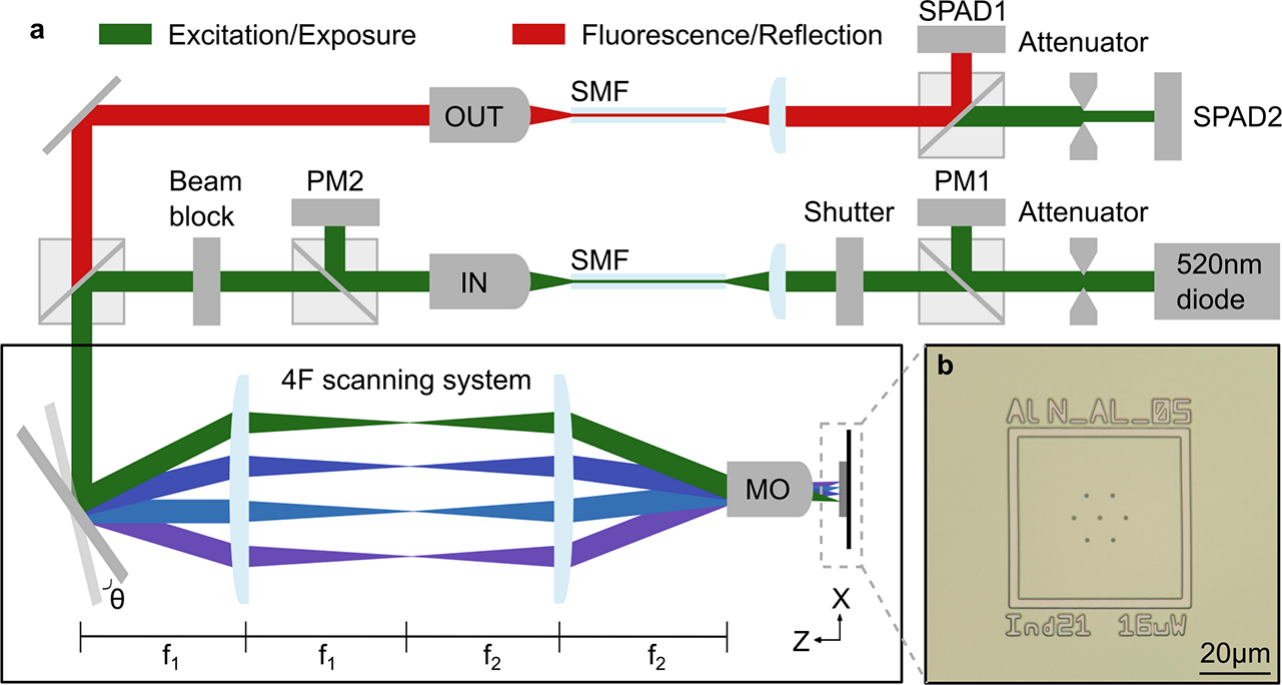
(a) Illustration of the laser-scanning confocal lithography microscope. The exposure/excitation path is highlighted in green. The fluorescence/reflection path is illustrated in red. The 4F scanning system is illustrated with four different colored beams, which represent the spatially overlapped excitation and fluorescence beams at different scan-mirror angles. PM = power meter, MO = microscope objective, SMF = single-mode fiber, and SPAD = single-photon avalanche diode. (b) Demonstration of an exposure in a resist bilayer, patterned with the scanning confocal lithography microscope in panel (a), before deposition and lift-off of metallic alignment markers for localizing the position of QEs.

Initially, the LSCL microscope is used to pattern nickel alignment markers, as illustrated in Figure 1b, through the use of a standard photoresist bilayer, metal deposition, and lift-off. Thereafter, the LSCL microscope simultaneously images the reflection of the laser and the resulting fluorescence from QEs. The reflection/fluorescence is coupled into and out of single-mode fiber (SMF), split onto two single-photon sensitive avalanche diodes (SPADs). The spectral window of 550–650 nm is detected on SPAD1, and the reflection of the 520 nm laser is detected on SPAD2. Identical optics in the excitation and collection path results in near-identical reflection point-spread functions (PSFs), which in turn minimizes the effective reflection PSF for diffraction limited imaging of the markers. The microscope has a theoretical full-width half-maximum (FWHM) focal spot size of 295 nm for a 520 nm laser focused through the 0.9 numerical-aperture microscope objective.^33^ The expected minimum feature size for the lithography is approximated by twice the 1/e^2^ Gaussian radius at the focus with 2*w*_0_ = 501 nm. The fluorescence (reflection) imaging resolution can be estimated by considering the confocal intensity PSF,^33^ with a resolution of 236 nm (214 nm).

## Quantum Emitter Localization

Next, we simultaneously measure the fluorescence from the QEs and the reflection of the alignment features to determine the approximate positions of the QEs, which are observed as point-like features in Figure 2b. The hexagonal arrangement of markers can be observed in the simultaneous reflection image shown in Figure 2a. The measurement demonstrates the near-diffraction-limited image of the alignment markers. The central marker, MK0, was chosen as the reference marker and has an FWHM size in *X* and *Y*, extracted from a two-dimensional Gaussian function (2dGF) fit, of 520 ± 20 and 570 ± 20 nm, respectively. All metal markers are patterned with the same dose and have the same size. The near-diffraction-limited marker size enables determination of the position of each marker from a 2dGF fit with minimal uncertainty. Thirteen QEs are highlighted in Figure 2b. The fluorescence image of each point-like QE is a direct measurement of the fluorescence PSF of our microscope, where a single image of QE3 has a FWHM of 386 ± 5 and 335 ± 5 nm in *X* and *Y*, respectively.

**Figure 2:**
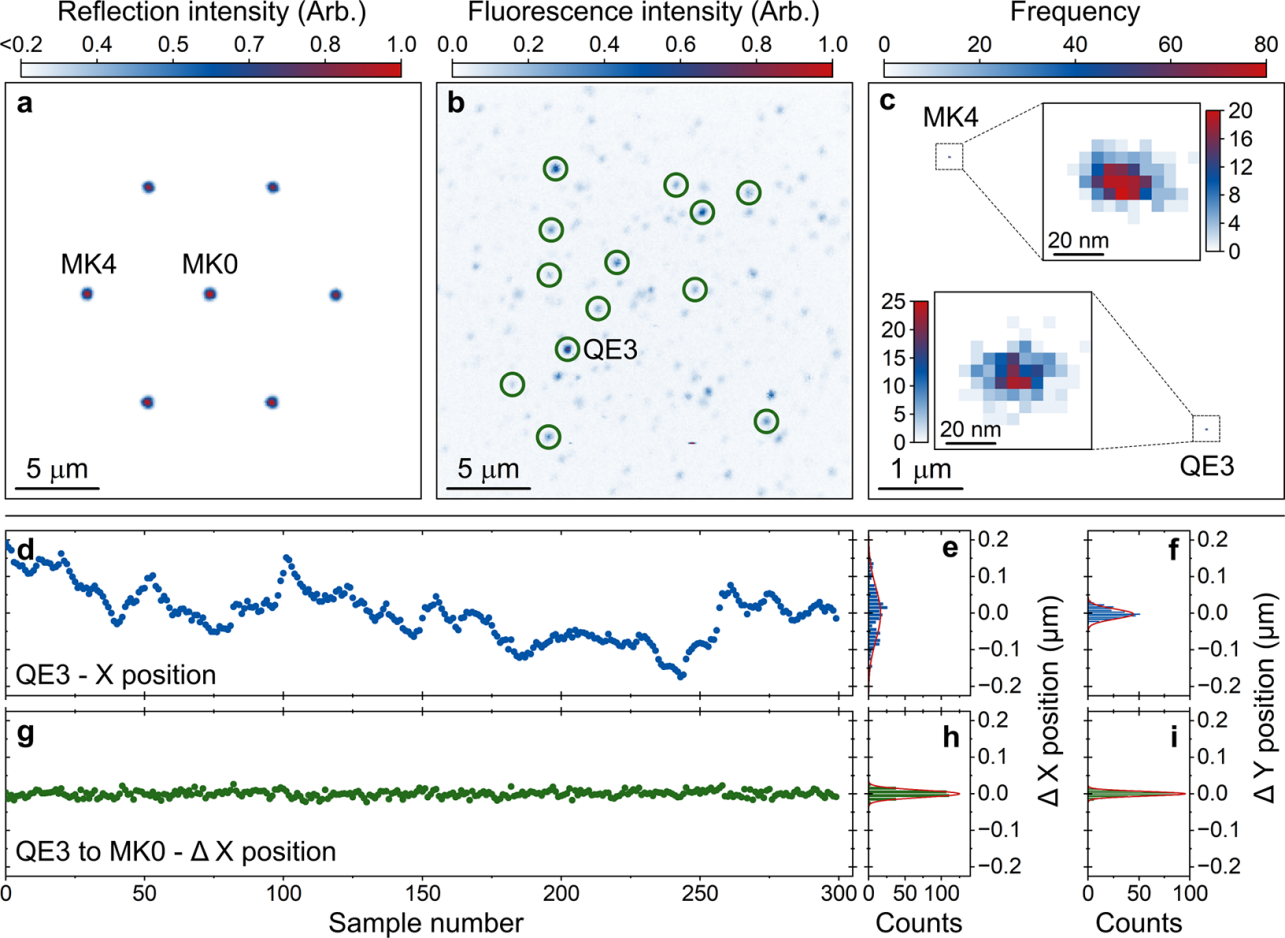
Drift-compensated localization of QEs in AlN. (a) A 25 × 25 μm reflection image of the near-diffraction-limited metallic markers lithographically deposited on the sample surface and (b) simultaneous fluorescence image revealing the approximate location of QEs. Thirteen QEs are highlighted (green, circles) in the image. (c) A bivariate histogram of the center point, *X*_c_ and *Y*_c_, over *N* = 300 repeat measurements of the positions of the emitter QE3 and the marker MK4, relative to MK0. The insets highlight the relative localization of QE3 and MK4. (d) Absolute *X* position of QE3 extracted from *N* = 300 repeat measurements, with an acquisition time of 4 s for each sample. Histograms in panels (e and f) show the binned *X* and *Y* positions of QE3, respectively, over the repeat measurements. (g) Relative *X* position of QE3 with respect to MK0 over *N* = 300 repeat measurements. Histograms in panels (h and i) show the binned relative *X* and *Y* positions of QE3 relative to MK0. The scatter plots in panels (d and g) share the *Y* axis of the histograms of panels (e, h).

The absolute position of the markers (emitters) can be determined by considering the *X* and *Y* center points, *X*_c_ and *Y*_c_, from a 2dGF fit to a reflection (fluorescence) image. For a single sample of marker MK4, highlighted in Figure 2a, we determine *X*_c_ and *Y*_c_ with fit uncertainties of δ*X*_c_ = 3.5 and δ*Y*_c_ = 3.3 nm. For a single sample of QE3 in Figure 2b, the uncertainties are δ*X*_c_ = 1.8 and δ*Y*_c_ = 1.6 nm.

We note that the extracted positions of the emitters and markers are affected by drift in the optical system over the long acquisition timescales (20 min) of the 2D images in Figure 2a,b, most of which is spent scanning areas with no features. The absolute position in *X* over *N* = 300 repeat measurements of the emitter QE3 is presented in Figure 2d, with a 4 s acquisition time per sample. Each repeat sample represents the extracted center point from a 2dGF fit to a 0.8 μm^2^ fluorescence image of the emitter. One can observe the deviation around the mean position from the histogram in Figure 2e,f, illustrating the drift in *X* and *Y*, respectively. Each histogram has a normal distribution, with two-standard-deviation (2STD) widths of *w*_*x*_ = 150 and *w*_*y*_ = 27 nm. We note a difference in drift between *X* and *Y*, which may be due to different mechanical moments of our sample stage and/or microscope objective mounting.

To overcome the positioning drift, we propose a repeated rapid sampling measurement, similar in nature to single-molecule localization microscopy (SMLM),^34^ to localize both the QEs and markers' position relative to a reference marker. By repeatedly determining the position of the emitter relative to the reference marker, with image acquisition times of 4 and 2 s for the emitter and marker, respectively, we eliminate long-timescale drift but retain the high photon numbers required to localize the emitter to within a fraction of the diffraction limit.

The relative *X* position difference between QE3 and MK0 over *N* = 300 repeat samples is illustrated in Figure 2g. Histograms of the *X* and *Y* position deltas can be observed in Figure 2h,i. A clear reduction in the distribution around the mean of the localized position of QE3 relative to MK0 can be seen from the data, where the 2STD uncertainty of a 1dGF fit to each histogram is reduced to *w*_*x*_ = 19.3 nm and *w*_*y*_ = 12.3 nm. The center point of the distribution, also extracted from the 1dGF fit of the histograms, can be determined with uncertainties of δ*X*_*c*_ = 140 and δ*Y*_*c*_ = 40 pm, representing subwavelength determination of relative emitter position. The bivariate histograms of the center point over 300 samples of MK4 and QE3 in Figure 2c illustrate the subdiffraction-limited localization of the relative positions of the markers and emitters.

## Emitter Projection Accuracy

We have shown that repeat sampling of QEs enables localization of their relative positions with subwavelength precision. We now quantify the expected LSCM lithographic patterning accuracy and precision. The sample is reloaded onto the microscope stage at an arbitrary orientation angle. The repeat-sampling algorithm is run on all seven marker positions to extract the rotation angle of the sample and the absolute position of MK0. The sample is iteratively reloaded, and the reference marker angle remeasured, until the sample angle closely matches the angle of the sample during the emitter localization algorithm. The patterning accuracy and precision are quantified by comparing a projection of the QE position with a measurement of the actual QE position for 38 QEs, which have been previously localized with the repeat-sampling algorithm.

For each of the 38 QEs sampled, we first determine the position of MK0 with a single reflection measurement and project the expected position of the QE, *P*_expected_. The projected position is compared with a measurement of the QE position, *P*_measured_, which is extracted from a 2dGF fit to a single 1 μm^2^ fluorescence image around *P*_expected_. The projection error, Δ*P*_e_(*x*, *y*) = *P*_measured_ – *P*_expected_, of the 38 QEs is presented in the scatter plot in Figure 3a.

**Figure 3:**
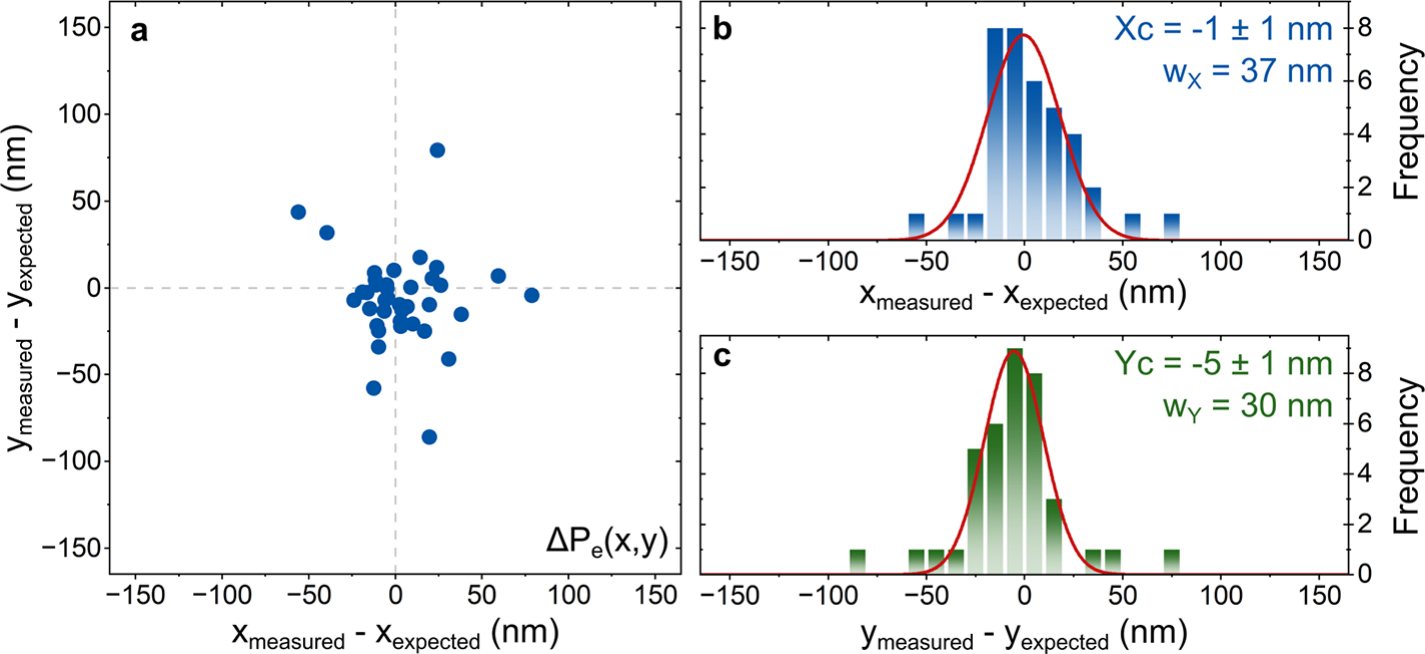
Statistical analysis of the optical lithography position accuracy and precision of 38 QEs. (a) Scatter plot of the *X* and *Y* projected position error, Δ*P*_e_(*x*, *y*). The error represents the positional delta between the projected and measured QE position. (b, c) Histograms illustrate the projected position error in *X* and *Y*, respectively. A 1dGF fit to each histogram is used to determine the normal distribution, where the center points (*X*_c_, *Y*_c_) and 2STD widths (*w*_*X*_, *w*_*Y*_) are illustrated.

The *X* and *Y* projection delta histograms are presented in Figure 3b,c revealing QE projection deltas consistent with normal distributions. The *X* and *Y* distributions have 2STD widths of *w*_*X*_ = 37 nm and *w*_*Y*_ = 30 nm, illustrating the patterning precision, which is dominated by the repeatability of the galvanometric stage used to control the beam position. Both the *X* and *Y* distributions are centered within a few nanometers of zero, demonstrating the patterning accuracy. We note that the outliers with δ*P*(*X*, *Y*) > (*w*_*X*_, *w*_*Y*_) correspond to QEs with a low signal-to-noise ratio.

## Spatially Aligned Lithography

We demonstrate the deterministic enhancement of QEs in the solid state using spatially aligned photonic microantennas in the form of etched semiconductor pillars. The micropillars act to guide the fluorescence toward the collection optics, overcoming losses associated with total internal reflection due to the large semiconductor-to-air refractive index contrast (*n*_AlN_/*n*_air_ = 2.15). Finite-difference time domain simulations were used to quantify the expected fluorescence enhancement over the 550–650 nm spectral window.

The lithographically defined micropillars were patterned with the custom LSCL microscope. To pattern the micropillars, we repeat the process previously used for the emitter projection error analysis in Figure 3 after the sample is coated with a bilayer photoresist stack. The positions and angles of the reference markers are localized through the resist stack without polymerizing the photosensitive resist layer. Once the marker positions and angles have been determined, we project the relative position of the 13 QEs illustrated in Figure 2b from MK0. Each emitter position is patterned with an optimized dose, stabilized using a software-defined control loop, to define a 1 μm aperture in the photoresist. The pattern is then transferred into the semiconductor using a deposited nickel mask, lift-off process, and dry etch.

A false-color scanning electron microscope (SEM) micrograph of the etched, spatially aligned micropillars is shown in Figure 4a. The hexagonal arrangement of the alignment markers, highlighted in red, can be observed. The spatially aligned micropillars are highlighted in blue. We note that the micropillars have been fully masked during the etch and have a flat top surface. Due to the 1800 repeat measurements of the position of MK0 during the localization of the markers with the resist stack in situ, the size of MK0 post-etch is larger than the other markers due to unwanted polymerization of the resist, which masks the semiconductor during the etch. This has no impact on the localization or lithographic accuracy. Striations can be observed on the sidewalls, which have an angle of θ = 76°. Optical fluorescence and reflection measurements of the spatially aligned micropillars are presented in Figure 4b,c, respectively. All 13 of the micropillars demonstrate fluorescence from QEs at their centers. The reflection image in Figure 4c illustrates an optical interference pattern when imaging the micropillar reflection due to the imaging systems being focused near the bottom of the micropillars.^8^

**Figure 4:**
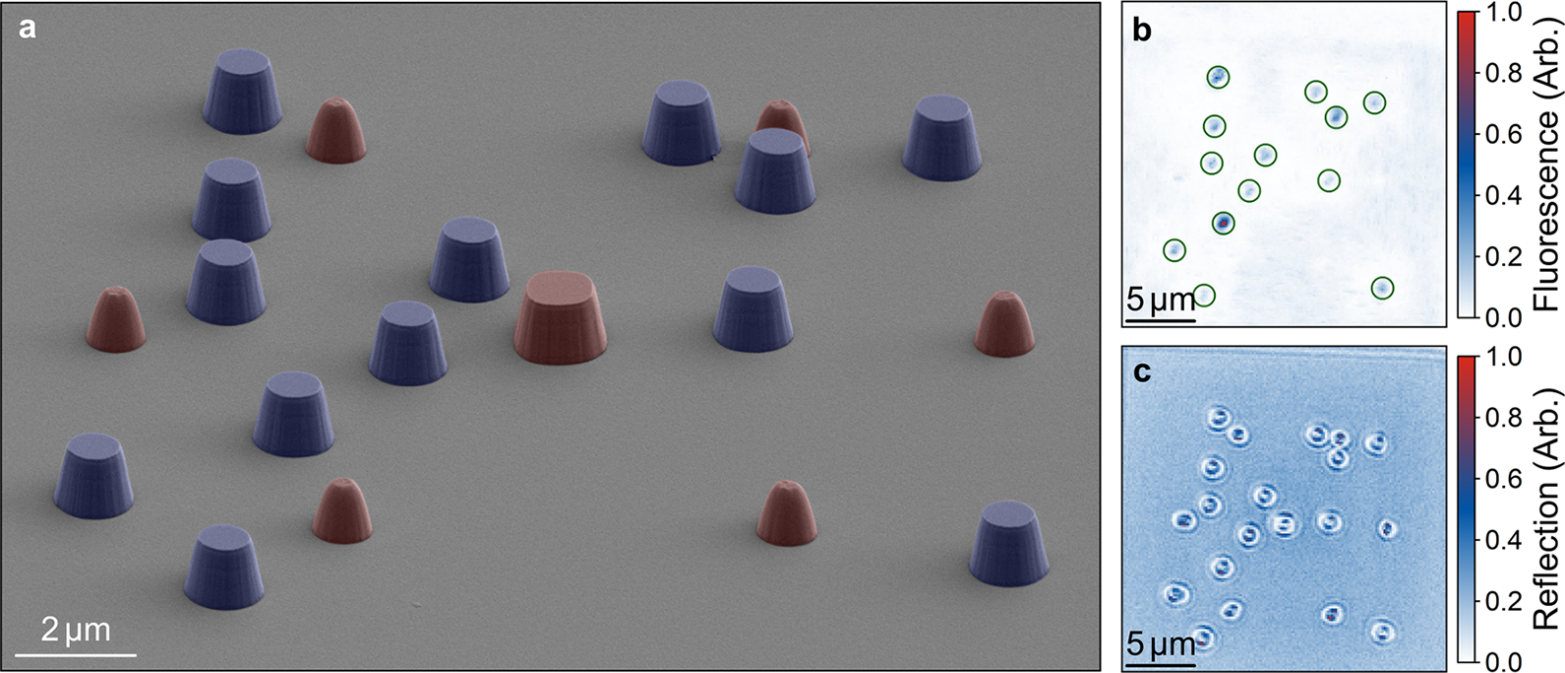
(a) False-color scanning electron micrograph of spatially aligned micropillars in AlN. The etched alignment markers are highlighted in red, and the QE micropillars are highlighted in blue. (b) Fluorescence image of the QEs embedded in micropillars. (c) Corresponding reflection image of the micropillars.

To quantify the enhancement of the QEs, we focus on each micropillar in turn. A power-dependent fluorescence intensity measurement is taken for each micropillar and compared to a measurement taken on the same emitter before patterning. Not all QEs showed an enhancement in the extracted photon rate. We note that the geometry of the micropillar was optimized to maximize the extracted light within the 550–650 nm spectral window, and the same design was exploited for each pillar. To make the enhancement more uniform, one would need to design the optical antenna considering the emission spectrum or each emitter, the emitters’ depth in the semiconductor, and the out-of-plane polarization angle of each QE.

Figure 5 shows optical measurements of the QE3. The spatial alignment of the etched micropillar can be observed by comparing the simultaneous fluorescence and reflection measurements in Figure 5a,b. Excitation power-dependent fluorescence intensity measurements are shown in Figure 5c,d. The data is fit with *I*(*P*) = *I*_*∞*_ *P*_sat_/(*P* + *P*_sat_), where *I*_*∞*_ is the intensity at infinite pump power and *P*_sat_ is the saturation power.^3^ Comparing *I*_*∞*_ for QE3 before and after embedment in a micropillar, we determine a 48 ± 1% enhancement in the collected photon intensity. The measured photon intensity rate enhancement is lower than predicted from numerical simulations, where a maximum average 6-fold enhancement across the 550–650 nm spectral window is determined. We hypothesize that the discrepancy between simulation and measurement arises due to the unknown depth, polarization angle (assumed in-plane), and different spectral properties of the emitters. In the future, preselecting emitters can enable matching the spectral response of the patterned optical device to each emitter. Further details on the simulation can be found in the Methods section. We note that the largest measured enhancement is 84 ± 6% from QE12, as illustrated in Figure 5d.

**Figure 5:**
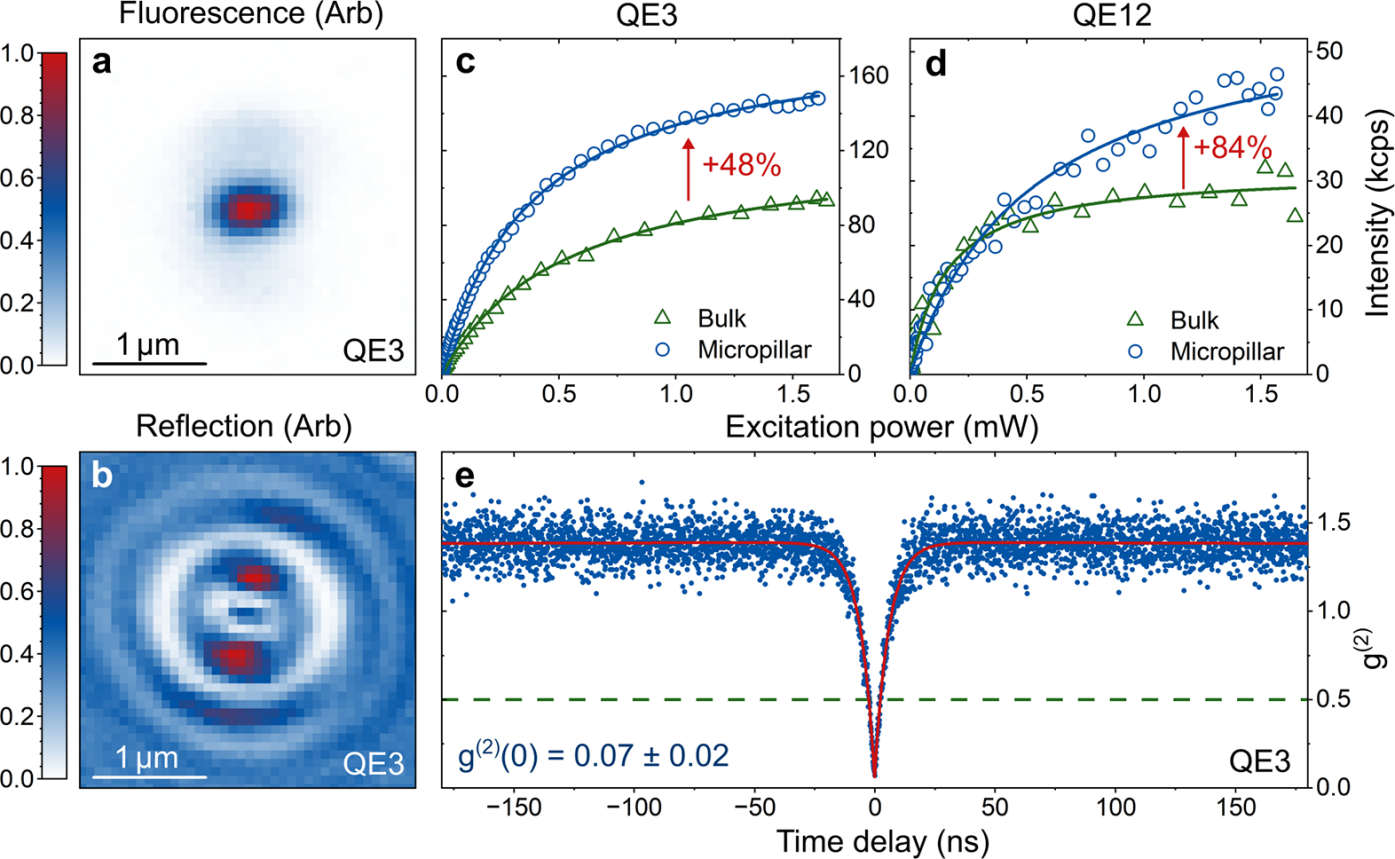
Optical properties of QE3. (a) Fluorescence image of QE3 in the micropillar. (b) Corresponding concurrent reflection image on the pillar. Both images are taken with optical focus on QE3. (c, d) Excitation power-dependent fluorescence intensity measurement of QE3 and QE12, respectively, before and after embedment in a micropillar. The measurements demonstrate 48 and 84% increases in the collected photon intensity at infinite pump power. (e) Second-order correlation measurement of QE3 in a micropillar, demonstrating antibunched photon statistics.

Figure 5e shows a second-order correlation measurement where clear antibunching can be observed, illustrating the quantum nature of the emission, with *g* ^(2)^(0) = 0.07 ± 0.02. In addition, bunching is observed on microsecond timescales, which is typical for QEs in AlN.^7^ The second-order correlation measurement is fit with a three-level energy model and normalized by considering *g* ^(2)^(τ) = *G* ^(2)^(τ)/*G* ^(2)^(*∞*).^3^ The normalization and fit to the second-order correlation in Figure 5e appear flat and offset from unity due to the microsecond timescale bunching.

## Discussion

The *X* and *Y* projection distributions in Figure 3 illustrate the accuracy and precision of the localization and patterning of the LSCL microscopy system. This represents a patterning accuracy of a few nanometers and a precision of a few tens of nanometers, an order of magnitude below the diffraction limit.

The precision is limited due to the repeatability of our current scanning stage and could be further improved with more repeatable galvanometric mirrors. In addition, one could increase the localization time with real-time monitoring of the stage position and increased reflected optical flux. This could enable multiframe-per-second sampling of the marker positions. A similar improvement in emitter sampling time could be achieved with integration of the emitter into a planar cavity, similar to what is achieved for InGaAs quantum dots.^17^

To enhance the patterning resolution, nonlinear optical lithography^32^ and/or electron-beam lithography could be used to define nanostructures with subdiffraction-limited feature size. Nevertheless, the ability to localize emitters with subwavelength precision and subsequently define nanostructures is a vital tool for increasing the efficiency and yield of bright quantum light sources.

## Methods and Materials

The QEs used in the paper to demonstrate the nanoscale localization microscopy and deterministic fabrication are room-temperature QEs in aluminum nitride (AlN). The AlN samples are cleaved from a 2 in. wafer of MOCVD-grown, 1 μm-thick, AlN-on-sapphire purchased from DOWA Electronics. The datasheet specifies a surface roughness of 1.6 Å over a 5 × 5 μm area, measured by atomic force microscopy in tapping mode. The emitters are of unknown origin but are readily present in the samples. Etch depth profiling experiments have demonstrated that the emitters reside within the first 75 nm of the AlN epitaxy.^8^ The optical properties are explored in detail in our previous works^7,8^ and typically have a zero-phonon line (ZPL) between 550 and 600 nm. The ZPL couples to phonons with a greater than 100 nm phonon sideband. Optical filtering between 550 and 650 nm is used for the power-dependent intensity measurements to quantify the brightness of the emitters pre- and postfabrication of micropillars.

All optical images used for the localization of emitters and marker positions are measured by displacing the excitation laser in *X* and *Y*, pixel-by-pixel, in a spiral pattern starting at the middle pixel. At each pixel, we dwell for 30–50 ms to measure the resultant fluorescence and reflection. The reflection of the laser is split from the fluorescence in the dichroic imaging system with a 550 nm long-pass dichroic mirror, with an additional 550 nm long-pass and 650 nm short-pass filter in the fluorescence path. Both the reflection and fluorescence are measured on single-photon-sensitive avalanche photodiodes from Excelitas.

To precisely control the optical dose at the resist during lithography, we use a series of optical power meters and beam blocks/shutters. The dwell time at each exposure pixel is controlled using a fast-modulating optical shutter, with on/off time scales of a few milliseconds. The optical power is stabilized before the lithography on the power meter “PM2”. During the power stabilization, the shutter is fully open and an optical beam block after the power meter is closed. This avoids unwanted polymerization of the resist. The laser power on PM2 is stabilized using a variable liquid-crystal attenuator and a software-defined control loop. Once the desired power is stabilized, the corresponding optical power is noted on PM1. The shutter is closed, and the beam block is opened, ready for exposure. For long-time scale exposures, the optical power is monitored and stabilized on PM1 during the exposure, as PM1 is located before the fast shutter. This minimizes optical elements after the spatial filtering of the single-mode-fiber coupling to achieve near-diffraction-limited optical patterning of the resist.

The metallic markers are fabricated using the custom LSCL microscope. A bilayer resist stack, comprising 600 nm of Shipley S1805 on top of 600 nm of LOR3A from MicroChem, is spin-coated and baked onto a cleaned sample of AlN. The LOR3A is baked at 200 °C for 5 min, and the S1805 is baked at 130 °C for 3 min. Both resists are spun at 6000 rpm. Holes are exposed in the resist-stack and developed in Shipley Microposit MIF319 developer for 1 min. The dose is optimized so that the diameter of the holes approaches the resolution limit of the 520 nm exposure laser with an NA = 0.9 microscope objective. The dose corresponds to a pixel dwell time of 10 ms and an optical power of 10 μW measured at PM2. After development, 100 nm of nickel is deposited using a thermal evaporator, and finally lift-off with *N*-Methyl-2-pyrrolidone (NMP) and a standard solvent clean. The 100 nm of nickel provides a high reflection of the 520 nm laser light during positioning of the markers, demonstrating a high marker-to-AlN reflection contrast of greater than 10:1. We note that the micron of AlN with a refractive index of 2.1 provides constructive thin-film interference; therefore, the signal-to-noise ratio could be further increased by thinning the epilayer, decreasing the reflection at the AlN-to-air interface.

To pattern the micropillars, the same metal lift-off process is used to define the markers. The S1805/LOR3A bilayer is spun onto the sample, which is subsequently reloaded into the custom LSCL microscope. The positions and angles of the markers are localized through the resist stack. The projected emitter locations are patterned with an optimized exposure time and optical power, and the resist is developed in MIF319 and deposited with 100 nm of Nickel. After lift-off, the sample is etched in an Oxford Instruments PlasmaPro 100 inductively coupled plasma (ICP) reactive-ion etcher (RIE). The gas chemistry is BCl_3_/Cl_2_/Ar with flow rates of 6/36/5 sccm. The table temperature is 20 °C with an ICP power of 550W and an RIE power of 80W. The chamber is at a pressure of 3.8 m Torr.

Finite-difference time-domain simulations were performed in Ansys Lumerical. A simulation environment was set up with electric-field monitors spanning a 1 μm micropillar of AlN on a sapphire substrate, with a unity background index. The pillar height remained constant during the simulations. The radius of the micropillar was swept to obtain the highest average collection efficiency from a dipole source positioned at the center of the micropillar, 75 nm from the sapphire substrate. The collection efficiency is determined by projecting the near-field radiation profile into the far field and integrating across a numerical aperture of 0.9. The radius sweep enabled optimization of the pillar dimensions to obtain the highest enhancement factor. The simulations are compared to the simulated collection efficiency of a dipole in a planar AlN thin film. We note that the simulations did not include the sidewall profile of 76° or a finite dipole out-of-plane orientation. We obtain an average collection efficiency enhancement of 6 over the 550–650 nm range, for a micropillar with a diameter of 1 μm. We note that a Purcell factor of near unity is determined from the simulations for a 1 μm micropillar, with an average value of 0.97 across the 550–650 nm spectral window. Further details with regard to the simulations can be found in the referenced conference proceedings.^35^

## Data Availability

Data supporting the findings of this study are available in the Cardiff University Research Portal at 10.17035/cardiff.31016893.
